# Manipulating free will beliefs using online video games

**DOI:** 10.1007/s00426-023-01815-x

**Published:** 2023-03-26

**Authors:** Nel Tavernier, David Wisniewski, Marcel Brass

**Affiliations:** 1 Berlin School of Mind and Brain/Department of Psychology, Humboldt-Universität zu Berlin, Luisenstraße 56 Haus 5, 10117 Berlin, Germany; 2 Department of Experimental Psychology, Ghent University, Ghent, Belgium; 3 Science of Intelligence, Research Cluster of Excellence, Berlin, Germany

## Abstract

Research in social psychology and experimental philosophy has investigated lay people’s free will beliefs (FWB). Using different approaches (i.e. experimental manipulations and vignette studies), they investigated how FWB relate to other concepts, and whether changing FWB has an impact on downstream processes such as social behavior. However, both approaches have shortcomings. While experimental manipulations used in social psychology suffer from demand effects, vignettes used in experimental philosophy are often highly abstract. Across two pre-registered studies, we developed a new approach by merging them in an online video game setting. Using this novel, experience-based FWB manipulation, we found that decreasing FWB impacted variables such as perceived control and responsibility in both studies. While the experience-based manipulation influenced participants’ beliefs in free will within the context of the experience (“Within the context of the scenario, would the agent believe in free will?”) in the first study, this manipulation effect did not transfer to participants’ general FWB (“Do you believe in free will?”) in the second study. Overall, our findings suggest a way forward in studying laypeople’s beliefs in free will.

## Introduction

The question of whether free will exists or not has been debated in philosophy for centuries, with competing views of various accounts (Dilman, 1999). More recently, cognitive neuroscientists and psychologists have tried to address this question empirically. Several studies found that voluntary actions are preceded by specific patterns of neural activity several hundred milliseconds before awareness (Fried et al., 2011; Haggard & Eimer, 1999; Libet et al., 1983, 1993; Soon et al., 2008). This observation has led some neuroscientists to the assertion that conscious free will is nothing more than an illusion (Crick, 1994; Harris, 2012; Libet et al., 1983). Although this viewpoint received various criticism (Brass et al., 2019; Radder & Meynen, 2013; Roskies, 2010; Schurger et al., 2021), it has become a popular topic in public media (Burkeman, 2021; Cave, 2016; Nichols, 2011). While lay people generally believe that free will exists, these anti-free will messages might challenge their beliefs in free will. In philosophy, there are competing ideas about the impact of disbelief in free will. Some philosophers have argued that disbelief in free will could have a severe negative impact on moral behavior (Smilansky, 2000, 2002), while others argue that it could have a positive impact on behavior, such as a decrease in retributivism (Shaw et al., 2019). Given these theoretical controversies, it is important to investigate what impact changing lay people’s beliefs in free will really has.

### Previous approaches investigating free will beliefs

In recent literature, there have been two streams of research investigating the impact of changing lay people’s beliefs in free will, namely social psychology and experimental philosophy (Nahmias et al., 2014; Vohs & Schooler, 2008). Within social psychology, researchers have used direct experimental manipulations to investigate the causal effects of FWB on downstream processes (Baumeister et al., 2009; Sharrif et al., 2014). Past research mainly used the so-called Crick method as an experimental manipulation technique, first introduced by Vohs and Schooler (2008). Participants were randomly assigned to an anti-free will group or a control group, where they either read a passage arguing against the existence of free will (i.e. a passage from “The Astonishing Hypothesis” by Francis Crick (Crick, 1994)), or a passage about consciousness without mentioning free will (Vohs & Schooler, 2008). After reading the text, a dependent variable is measured (e.g. prosocial behavior), and a self-report questionnaire on free will beliefs is administered as a manipulation check (Ewusi-Boisvert & Racine, 2018). These studies found that inducing disbelief in free will increases antisocial behaviors (Baumeister et al., 2009; Protzko et al., 2016; Vohs & Schooler, 2008), have an impact on cognitive factors (Rigoni et al., 2015), and impacts ideas of morality and justice (Sharrif et al., 2014).

Alternatively, in experimental philosophy, hypothetical vignette studies are employed to investigate laypersons' beliefs in free will. In these studies, researchers create scenarios in which they sketch a world where a certain person has to make a decision, which can either be in their own control (i.e. free condition) or out of their own control (deterministic condition). Afterward they are asked questions regarding attributions of free will within the context of this scenario (e.g. responsibility, moral behavior) (Nahmias et al., 2014; Shepard & Reuter, 2012; Shepherd, 2012). Here, instead of using a direct experimental manipulation, this approach creates a hypothetical scenario. Additionally, instead of measuring general FWB, FWB are measured within the specific context of the vignette and investigate how these relate to other concepts.

Both approaches have their pros and cons. An advantage of both approaches compared to correlative approaches is that they use experimental manipulations and therefore allow to draw causal inferences. The social psychological approach, however, has been heavily criticized because key findings could not be replicated (Eben et al., 2020; Genschow et al., 2020, 2021; Nadelhoffer et al., 2020). A recent meta-analysis showed that while free will belief manipulations have a reliable effect on free will beliefs as measured by questionnaires, downstream effects on other variables such as social behavior were primarily driven by underpowered studies (Genschow et al., 2021). Furthermore, the question arises whether effects on free will related beliefs questionnaires are due to demand effects (Mummolo & Peterson, 2019). After all, it is difficult to imagine that reading a short text changes a complex belief system that has been acquired over a lifetime (Tavernier, Wisniewski, Brass, in prep.). The main advantage of the experimental philosophy approach is that it does not rely on changing free will beliefs. By creating a hypothetical scenario, it only requires that participants are willing to imagine themselves in this scenario. This is of course also its weakness. Vignette studies often employ highly hypothetical, counterfactual, and far-fetched scenarios, thus relying heavily on the imagination of the participant. Furthermore, the approach does not investigate how people behave but only how people imagine to behave.

To conclude, the FWB literature is still in need of an effective method of manipulation, which allows us to investigate the relation between FWB and downstream processes. The social psychology approach does not provide such an effective method of manipulation as these studies fail to replicate an impact on downstream processes, and consist of demand effects and complex philosophical arguments. Moreover, the experimental philosophy approach does not provide such an effective method either as these focus on hypothetical scenarios that are far-removed from real-life behavior, and a transfer to general beliefs outside the context of the vignette is typically not assessed.

The goal of the current study is to overcome some of the limitations of the two outlined approaches by merging both approaches in an online gaming setting. Such a setting enables us to let participants experience a reduction of free will directly, and creates a FWB manipulation that is not based on understanding complex philosophical arguments or counterfactual scenarios. We know from past research that agency and FWB are closely related (Aarts & van de Bos, 2011; Feldman, 2017; Lynn et al., 2014), suggesting that experiencing limited agency over actions and outcomes decreases beliefs in free will. Therefore, we implemented the manipulation of reduced agency in the low free will scenarios. Similar to the social psychology approach, we used a direct experimental manipulation instead of a hypothetical manipulation as in the experimental philosophy approach. By doing so, we could investigate participants’ beliefs and behavior. Importantly, we replaced the text-based manipulation by an experience-based manipulation of reduced agency. However, similar to the experimental philosophy approach, we create a hypothetical scenario in which participants experience this reduction of agency. We hypothesized that experiencing reduced agency over one’s own action and their outcome would decrease beliefs in free will (Hypothesis 1) and increase beliefs in determinism (Hypothesis 2). Furthermore, we investigated whether the manipulation of reduced agency impacted FWB within the context of the scenario, and whether this transferred to participants’ general beliefs (*Study 1 vs. Study 2).* Combining the social psychology approach and the experimental philosophy approach opens up new opportunities to study free will beliefs and their impact on downstream processes, with ample room for refinement of the FWB research methodology.

### The current study

Across two pre-registered studies with a total sample size of 599 participants, we created a novel FWB manipulation technique by merging experimental manipulation and vignette techniques. We described a scenario similar to a vignette study, but then, letting participants experience the described scenario directly instead of just letting them imagine it. Across 3 conditions, participants played online videogames where they experienced more or less agency over actions and outcomes. In a first study, similar to the experimental philosophy approach, we investigated whether this experience affected free will and determinism beliefs within the context of the online video game (“Within the context of the scenario, would the agent believe in free will?”). Similar to the social psychology approach, we manipulated FWB directly. Thus in a second study, we investigated whether this experience of reduced agency over actions and outcomes affected general free will and determinism beliefs (“Do you believe in free will?”).

## Study 1 contextualized

## Method

### Open science statement

We report all measures, conditions, data exclusions, and how we determined our sample size. Questionnaires, data, analysis scripts, and Unity scripts are available on the Open Science Framework (OSF; https://osf.io/xh36s/). All confirmatory and exploratory analyses are pre-registered on OSF (https://osf.io/bsf8w).

### Participants

We performed a Bayesian sequential sampling plan to determine the required sample size to detect the effects of our FWB manipulation. We started with 50 participants in each condition (total *n* = 150). Our primary measure of manipulation strength was the contextualized free will scale of the FWI. We performed two one-sided Bayesian Independent sample t tests (BayesFactor package in R; default priors) to compare the conditions (free choice > limited choice; free choice > no choice; for details on the design and measures see below). In case of inconclusive evidence (0.3 < BF10 < 3), we increased the sample size by 10 per group until either evidence was conclusive (BF10 > 3 or BF10 < 0.3) or we reached the cut-off of 100 participants per group (*n* = 300 in total).

All participants were recruited and paid online through the Prolific platform. The inclusion criteria consisted of first or fluent language: English, age between 18 and 40, at least 10 prior submissions on the platform, and at least a 70% approval rate. Participants’ complete dataset was excluded when: (a) their total score of the manipulation game was lower than the mean score of the condition they were in—2.5 times the standard deviation. (b) the total duration of the experiment exceeded the second quartile (75%) + 1.5 * IQR, calculated from all the durations of all participants. (c) they took less than 300 ms (per item on average) to complete the FWI, as this would mean that they did not read and answer all the statements properly.

The final sample consisted of *n* = 149 in total (free choice *n* = 50, limited choice *n* = 48, no choice *n* = 51). The mean age was 28.24 (range 18–40 years; *SD* = 6.45); 64.9% of the sample was female, 33.8% was male, and 1.3% selected other.

### Procedure

The experiment was conducted online in a web browser using Jatos (v. 3.6.1). At the beginning of the experiment, participants could read a document containing general information regarding the procedure and then gave informed consent. They were told that the purpose of the study was to investigate user experiences in video games. At the end of the experiment, they received a full debriefing of the purpose of the study.

#### Demographic data

Participants were first asked to provide demographic information. They were asked about their age and gender (male, female or other).

#### Games

**Fig. 1:**
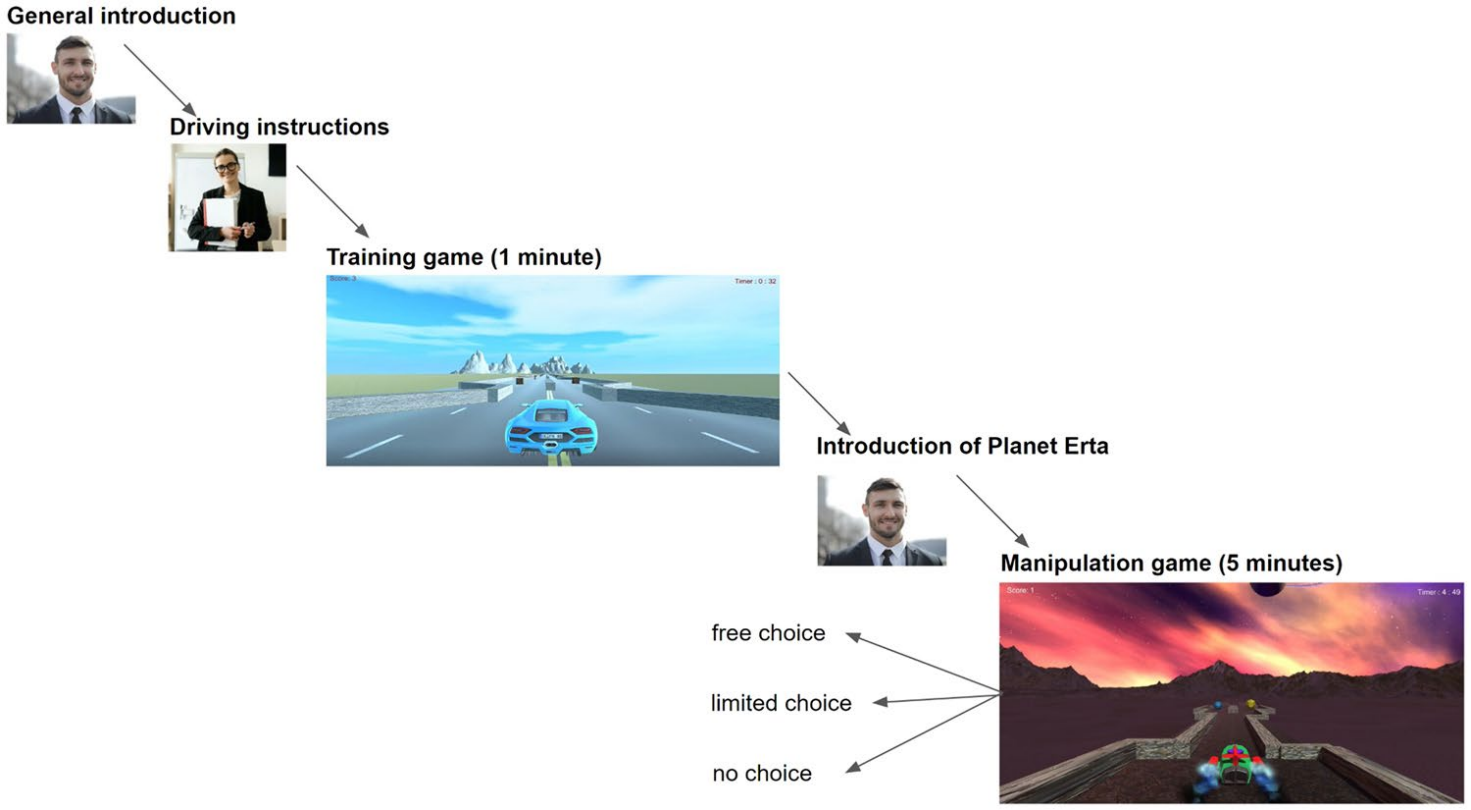
Structure of the two online video games. **a** General introduction, **b** Driving instructions, **c** Training game of one minute (same for all conditions), **d** Introduction of Planet Erta, **e** Manipulation game of five minutes (free choice vs. limited choice vs. no choice condition)

Next, participants were instructed to play two games in total, a training game, and a manipulation game. After piloting the study, we chose the duration of both games based on preliminary results and participants’ feedback (training game = 1 min; manipulation game = 5 min). Both the video games were developed in Unity (v. 2021.3.1f1). Further, we programmed the game design using C# in Rider (Jetbrains, v. 2021.3) and implemented these scripts into Unity. These games were then exported to a WebGL and implemented into Jatos. Figure 1 shows the step-by-step procedure of the games. The games can also be found on OSF (https://osf.io/xh36s/). Below, both games are discussed in more detail.

##### Training game

Participants had to play a video game for 1 min, which was identical for all three conditions. First, they saw a general introduction screen which explained to them that they will have to work for a company called *‘Logistics and Intergalactic Transport’* (LIT). They were told that they will work as an intergalactic courier driver, and their job is to pick up as many packages as possible that needed to be delivered to a central warehouse. Then we told them that they first will be sent to a training camp in the United States of America, where they will learn all the skills to become a professional pick-up driver, and afterward they will be sent off-world to the *Andromeda Galaxy*. After reading this general introduction, they received instructions on how to drive the car. Using the arrow keys, they could choose to drive forward, left or right, by pressing one of the three keys. To pick up a package, they just had to drive towards it, and it got picked up automatically, giving them a + 1 score. When starting the game, they had to drive through forked roads. From the starting point, participants had to drive 8 s to reach the first crossing. At every crossing, they had to make a choice to pick up one of two packages, by driving to the left or right fork. After making this decision between the left and right fork, participants had to drive 3 s to pick up the package. After picking up a package, participants had to drive 6 s to get to the next crossing. Within the time limit of 1 min, participants could pick up a maximum of eight packages. After 1 min, they got a ‘Game Over’ screen and they got redirected to the manipulation game.

##### Manipulation game

Subsequently, participants had to play a video game for 5 min, which differed for the three conditions. First, they saw an instruction screen that explained to them in more detail the world that they were sent to. They were told that they will be sent to *Planet Erta* in the *Andromeda Galaxy* and that on Erta the landscape and life are very similar to earth. We explained to them that on Erta there are these advanced life forms called Ertans, who look, talk, and behave very much as we do. After reading this introduction of Planet Erta, they could start playing the game. Although the visuals differed from the training game, they also had to drive through forked roads and decide at every crossing to select a package on the left or right fork. From the starting point, participants had to drive 6 s to the first crossing. After making the choice between the left and right fork, participants had to drive 3 s to pick up the package. After picking up a package, participants had to drive 10 s to get to next crossing. One key difference with the training game was that instead of using identical packages for the left and right fork, we opted for different shapes and colors, which made the choice between left and right less arbitrary. Depending on which condition they were in, they had more or less control over this choice. Specifically, agency over actions and outcomes were manipulated to let participants experience a loss of control. The *free choice* condition was identical to the training game, here participants could freely choose between going left or right to pick up a package. Thus in the *free choice* condition, participants had full agency over their actions and outcomes. In the *limited choice* condition participants had control over their action keys in the game, e.g. if they pressed the forward arrow key the car would move forward. Different from the free choice condition, invisible walls were placed on the road which did not allow them to go left or right. When they drove into the crossing point, they bumped into a wall, forcing them to pick up one of the two packages. Thus in the *limited choice* condition, participants did have agency over their actions, while they did not have agency over the outcome. In the *no choice* condition participants had no control over the action keys, i.e. whichever arrow key they pressed, the car followed a predetermined path. Consequently, participants had no choice over which package they would pick up. Thus in the *no choice* condition, participants neither had agency over their actions nor the outcomes. Within the time limit of 5 min, participants could pick up a maximum of 33 packages. After 5 min, they got a ‘Game Over’ screen and they got redirected to the questionnaires.

##### Features of the games

The usage of video games as an experience-based manipulation of FWB provides some distinct features as opposed to the traditional methods in FWB literature. As described by Juul (2005), a game can be defined by six different game features, some of which also apply to our manipulation. The first feature described by Juul (2005) is that a game consists of a rule-based formal system. In our manipulation, participants were instructed to drive through roads using specific keys and collect as much points as possible by bumping into crates, thus consisting a formal set of rules. Secondly, a game has variable and quantifiable outcomes, which corresponds to the score participants achieved by collecting crates. Thirdly, a game consists of an interactive part in which the player exerts effort to influence the outcome. Importantly, this interactivity is the major advantage to our newly developed FWB manipulation, as previous methods of manipulation merely focus on passive reading.

#### Questionnaires

After playing both video games, participants first had to fill in the contextualized FWI. We took the original version of Nadelhoffer et al. (2014), which has 15 statements in total, 5 about free will, 5 about determinism, and 5 about dualism, and these were presented in a random order (responses: 7-point Likert scale, 1 = strongly disagree, 4 = neither agree nor disagree, 7 = strongly agree). For the contextualization of this FWI we changed the words ‘people’ and ‘humans’ to ‘ertans’ (https://osf.io/7va4q/). We also added an instruction to the questionnaire which they had to read carefully before responding to the statements:

*“Please imagine now that you are living on this Planet Erta of the Andromeda Galaxy. Keeping this in mind, please indicate how much you agree with the following statements in this scenario. Try to give an answer on these statements based on the experience you had with this Planet Erta. There is no right or wrong answer. So even if you are not sure about your answer, nevertheless try to complete the questionnaire based on the knowledge you have.”*

After filling in this contextualized FWI, participants had to respond to several control questions. First, we asked them how difficult it was to be a pick-up driver on Planet Erta (responses: 7-point Likert scale, 1 = very easy, 7 = very difficult). Then, we asked them how much control they experienced over the car on Planet Erta (responses: 7-point Likert scale, 1 = no control at all, 7 = full control). Subsequently, we asked them how responsible they felt for their actions on Planet Erta (responses: 7-point Likert scale, 1 = I was not responsible at all, 7 = I was fully responsible). The order of these three questions was randomized. Next, they had to rate their experienced arousal and valence while being a pick-up driver on Planet Erta measured by the Self-Assessment Manikin (SAM scale, Bradley & Lang, 1994). Lastly, we added a general questionnaire regarding Immersive Tendencies (Witmer & Singer, 1996).

### Analyses

All confirmatory and exploratory analyses described here were performed using RStudio (v. 2022.02.0, R v. 4.1.3).

#### Hypothesis 1: does the FWB manipulation affect contextualized free will beliefs?

Our first main hypothesis was that the experience of reduced agency over action and outcome (i.e. having no choice) would decrease beliefs in free will. Specifically, we hypothesized that the mean free will score would be the highest in the free condition, already lower in the limited choice condition, and the lowest in the no choice condition. To test this hypothesis, we performed a Bayesian ANOVA with the 3 conditions (BayesFactor package in R). The default priors used for the Bayesian ANOVA and Independent samples *t* tests are described in Rouder et al. (2012). A Jeffries prior was placed on μ and σ^2. Independent scaled inverse-chi-square priors with one degree of freedom were placed on the g-priors. If the BF10 was ≥ 3, we performed Bayesian Independent samples t tests between the 3 conditions. The Bayesian ANOVA was our undirected omnibus test, and the t tests tested our directed hypothesis (FW: free choice > limited choice > no choice). To check for robustness, we assessed for each Bayesian t-test whether results would change if the *r* scale of the priors changed. The default *r* scale was √2/2, but we also used *r* = 1, and *r* = √2. Unless stated otherwise, results were robust to changes in priors.

#### Hypothesis 2: does the FWB manipulation affect contextualized determinism beliefs?

Our second main hypothesis was that this experience of reduced agency over action and outcome would increase beliefs in determinism. Specifically, we hypothesized that the mean determinism score would be the lowest in the free condition, already higher in the limited choice condition, and the highest in the no choice condition. To test this hypothesis, we performed the same analyses as for *Hypothesis 1*, with the Bayesian ANOVA as our undirected omnibus test, and the t tests as our directed hypotheses (DET: free choice < limited choice < no choice).

#### Exploratory analysis 1: does the FWB manipulation also affect contextualized dualism beliefs?

While in many previous experiments using FWB manipulations researchers mainly focus on free will and determinism beliefs, some studies have found a close relation between lay people’s free will and dualism beliefs (Wisniewski et al., 2019). To further explore this possibility, we performed the same analyses as outlined in *Hypothesis 1, 2,* replacing the dependent variable by the mean dualism score.

#### Exploratory analysis 2: control questionnaires

For the variables difficulty, control, responsibility, arousal and valence we performed the same analysis as outlined in *Hypothesis 1, 2.* By doing this, we could investigate how the experience of the manipulation game differed across conditions. Further, we explored how the Immersive Tendencies questionnaire could be related to the FWI subscales. For this purpose, we performed an exploratory regression plot between the mean IT score and the different FWI subscales.

#### Exploratory analysis 3: agentive behavior

We acquired behavioral data from both games, i.e. score of the games, which packages they picked up, x and y coordinates of the car, pressed keys, and press duration of these keys. We were particularly interested in how the agentive behavior of participants could differ across conditions in the manipulation game. Thus we wanted to investigate whether a decrease in agency (i.e. *limited choice* and *no choice* condition) would affect agentive behavior in the game. To explore this, we investigated the sum of the total amount of arrow keys participants pressed, i.e. the keys they used to move forward, left and right, across the conditions and the different minutes of the game. As a reminder, during both games participants could navigate the car using the up, left, and right arrow keys. For the purpose of this analysis, we performed a Bayesian two-way ANOVA with the factors condition (free vs. limited vs. no choice) and the time range of the manipulation game (minute 1 vs. 2 vs. 3 vs. 4 vs. 5). For this analysis, the same R package and default priors were used as described in* Hypothesis 1.*

## Results

### Hypothesis 1

**Fig. 2:**
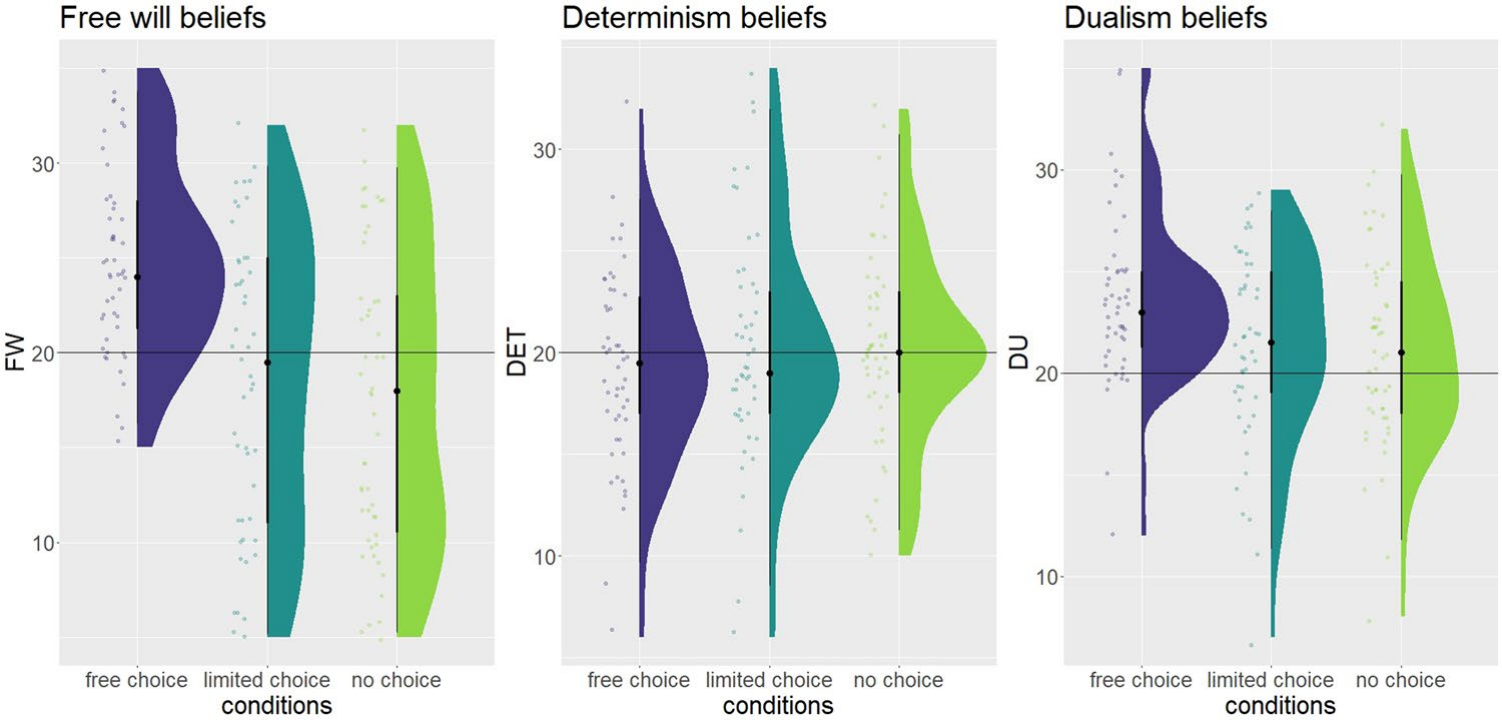
Responses to the contextualized subscales of the FWI (free will, determinism, and dualism). The horizontal line represents the midpoint of the scale, values below the line indicate disbelief in the statements, values above the line indicate belief in the statements. Each dot represents a single participant, data is jittered for display purposes

We expected the manipulation to affect free will beliefs, and found decisive evidence for a difference in the mean free will score between the three conditions (BF10 > 100, Fig. 2, Table 1). Further, the Bayesian Independent samples *t* tests revealed decisive evidence for the difference between the free choice and the limited choice condition (BF10 > 100), and for the difference between the free choice and the no choice condition (BF10 > 100). Substantial evidence against the difference between the limited choice and no choice condition has been found (BF10 = 0.25), which indicates no difference in beliefs in free will between these two conditions. As hypothesized, participants had a decreased belief in free will in the limited choice and no choice condition compared to the free condition. In contrast to our hypothesis, we have found no further decrease of free will beliefs in the no choice condition compared to the limited choice condition.

**Table 1:** Descriptive statistics. The mean (*M*) and standard error (*s.e.*) are shown for each subscale of the FWI (free will, determinism, and dualism), and for each control question (difficulty, control, responsibility, arousal and valence), separated for the three conditions

	FWB		DET		DU		Difficulty		Control		Responsibility		Arousal		Valence	
	*M*	*s.e*.	*M*	*s.e*.	*M*	*s.e*.	*M*	*s.e*.	*M*	*s.e*.	*M*	*s.e*.	*M*	*s.e*.	*M*	*s.e*.
Free choice	24.9	0.7	19.3	0.69	23.6	0.59	2.22	0.24	5.88	0.18	6.28	0.18	2.26	0.19	5.1	0.18
Limited choice	18.4	1.14	20.3	0.83	21.2	0.69	3.35	0.23	3.35	0.17	3.77	0.23	3.12	0.2	4.29	0.14
No choice	17.5	1.1	20.4	0.68	21.1	0.67	2.78	0.28	2.67	0.23	2.57	0.25	3.12	0.25	4.35	0.2

### Hypothesis 2

We expected the manipulation to affect determinism beliefs but found moderate evidence against a difference in the mean determinism score between the three conditions (BF10 = 0.12, Fig. 2, Table 1). Thus we have found no difference in determinism beliefs between the three conditions.

### Exploratory analysis 1

We wanted to explore whether the manipulation affects dualism beliefs, and found substantial evidence for a difference in the dualism subscale between the 3 conditions (BF10 = 3.56, Fig. 2, Table 1). Further, the Bayesian Independent samples t tests revealed substantial evidence for the difference between the free and limited choice condition (BF10 = 4.31), and for the difference between the free and no choice condition (BF10 = 4.93). Substantial evidence against the difference between the limited choice and no choice condition has been found (BF10 = 0.21), which indicates no difference in dualism beliefs between these two conditions. Thus participants had a decreased belief in dualism in the limited choice and no choice condition compared to the free choice condition.

### Exploratory analysis 2

We wanted to investigate how the experience of the manipulation game differed across conditions, by looking at perceived difficulty, control, responsibility, arousal, and valence. The descriptive statistics are shown in Table 1, and the results of the Bayesian ANOVAs and Bayesian Independent Samples t tests are shown in Table 2. These results indicated that participants perceived the manipulation game as more difficult in the limited choice condition compared to the free choice condition. Further, we found that participants felt more in control in the free condition compared to the limited and no choice condition, but no difference was found between the limited choice condition and the no choice condition. While for perceived responsibility, we did find evidence for a difference between all the three conditions (free choice > limited choice > no choice). Lastly, for perceived arousal and valence measured by the SAM scale, we found that participants were more negatively aroused in the limited and no choice conditions compared to the free choice condition. For the Immersive Tendencies questionnaire, we did not find any interesting results.

**Table 2:** Analyses for control questions. We computed Bayesian ANOVAs and Bayesian Independent samples t tests for every control question, and the Bayes Factors (BF10) are reported

Test	Difficulty	Control	Responsibility	Arousal	Valence
ANOVA	4.50	> 100	> 100	5.81	16.29
free choice-limited choice	33.65	> 100	> 100	14.71	41.53
free choice-no choice	0.46	> 100	> 100	4.30	4.91
limited choice-no choice	0.62	2.70	> 100	0.21	0.22

### Exploratory analysis 3

We wanted to explore how agentive behavior in the manipulation game would differ across conditions. The Bayesian two-way ANOVA indicated decisive evidence for a difference in agentive behavior between the three conditions (BF10 > 100, Table 3), and between the different time ranges (BF10 > 100), but strong evidence against the interaction between condition and time range has been found (BF10 = 0.05). Next, we performed post hoc two-way ANOVAs to compare the keypresses of the different conditions separately. For every combination of two conditions (free choice vs. limited choice; free choice vs. no choice; limited choice vs. no choice) we found decisive evidence for condition (BF10 > 100) and time range (BF10 > 100). Thus we found that agentive behavior decreased with the amount of agency participants have in the manipulation game (free choice > limited choice > no choice). And over all the conditions, we found that agentive behavior decreased when participants were proceeding further in the experiment.

**Table 3:** Descriptive statistics. The mean (*M)* and standard error (*s.e*.) of the total amount of keypresses in the manipulation game for every condition (free choice, limited choice, no choice), split by the time range (minute 1 vs 2 vs 3 vs 4 vs 5)

	Free choice		Limited choice		No choice	
	*M*	*s.e.*	*M*	*s.e*.	*M*	*s.e*.
1st min	62.40	8.96	55.30	2.09	43.20	6.48
2nd min	47.30	3.40	48.20	1.78	18.20	2.31
3rd min	42.90	2.36	46.40	1.74	15.00	2.00
4th min	43.60	2.32	46.70	2.15	13.70	2.39
5th min	42.40	3.17	43.20	1.98	10.80	1.94

## Interim discussion

This first study showed that within the context of the online game, our experience-based manipulation of reduced agency over action and outcome had an impact on free will and dualism beliefs, while it had no impact on determinism beliefs. Interestingly, the limited choice condition and the no choice condition did not differ regarding beliefs in free will and dualism. Further, we found that participants perceived less control and responsibility in the reduced agency conditions, and felt more negatively aroused. While the limited choice and no choice condition did not differ regarding free will beliefs, they differed regarding experienced responsibility. Lastly, our manipulation impacted participants’ agentive behavior in the game (i.e. data of key presses to move forward, left and right). These results align closely with the experimental philosophy literature. The question remains whether this effect transfers to general beliefs in free will, as suggested in the social psychology literature. To address this question, we set up a second study with an identical procedure as the first study, only replacing the contextualized version of the FWI with its canonical version to measure participants’ general FWB. By doing this, we could investigate whether our experience-based manipulation also impacted participants' own beliefs in free will.

## Study 2

### Method

#### Open science statement

We report all measures, conditions, data exclusions, and how we determined our sample size. Questionnaires, data, analysis scripts, and Unity scripts are available on the Open Science Framework (OSF; https://osf.io/xh36s/). All confirmatory and exploratory analyses were identical to study 1, and are pre-registered on OSF (https://osf.io/2rucd).

#### Participants

We performed a Bayesian sequential sampling plan to determine the required sample size to detect the effects of our FWB manipulation. We started with 100 participants in each condition (total *n* = 300). Our primary measure of manipulation strength was the free will beliefs scale of the FWI. We performed two one-sided Bayesian Independent sample *t* tests (BayesFactor package in R; default priors) to compare the conditions (free choice > limited choice; free choice > no choice; for details on the design and measures see Study 1). In case of inconclusive evidence (0.3 < BF10 < 3), we increased the sample size by 10 per group until either evidence was conclusive (BF10 > 3 or BF10 < 0.3) or we reached the cut-off of 150 participants per group (*n* = 450 in total).

All participants were recruited and paid online through the Prolific platform. The inclusion and exclusion criteria were identical to the first study.

The final sample consisted of *n* = 150 in each condition (*n* = 450 in total). The mean age was 29.15 (range 18—40, *SD* = 6.18); 65.7% of the sample was female; 33.8% was male, and 0.5% selected other.

#### Procedure

The procedure was identical to study 1, except for one key difference. We used the original FWI of Nadelhoffer et al. (2014), without contextualizing the statements. This allowed us to measure participants’ free will beliefs. Additionally, we removed the Immersive Tendencies questionnaire of Witmer and Singer (1996), as we did not find any interesting results in the exploratory analyses of the first study.

## Results

### Hypothesis 1

**Fig. 3:**
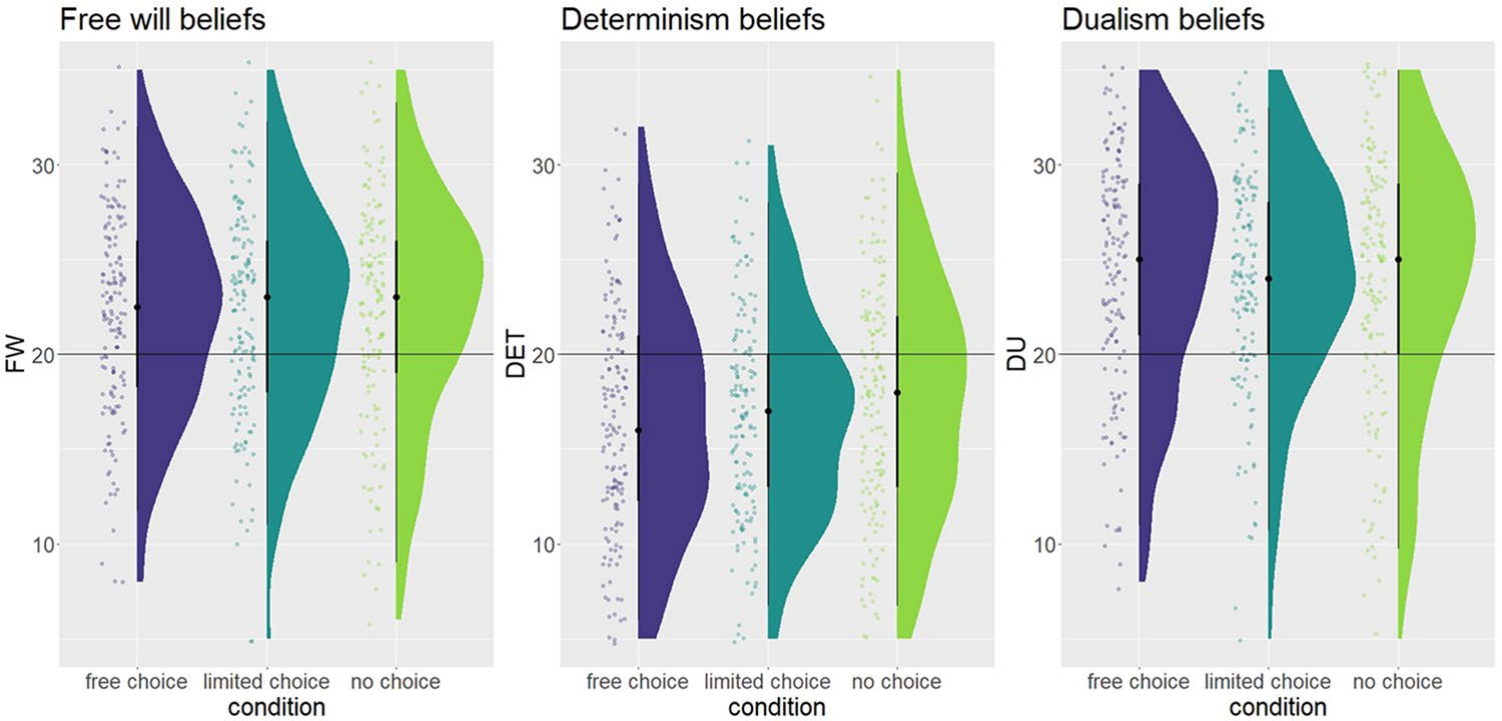
Responses to the subscales of the FWI (free will, determinism, and dualism). The horizontal line represents the midpoint of the scale, values below the line indicate disbelief in the statements, values above the line indicate belief in the statements. Each dot represents a single participant, data is jittered for display purposes.

We expected the manipulation to affect free will beliefs. Using a Bayesian ANOVA, the Bayes factor indicates very strong evidence against an effect of condition (BF10 = 0.026, Fig. 3, Table 4). Thus we have found no difference in free will beliefs between the three conditions.

**Table 4:** Descriptive statistics. The mean (*M*) and standard error (*s.e.*) are shown for each subscale of the FWI (free will, determinism, and dualism), and for each control question (difficulty, control, responsibility, arousal and valence), separated for the three conditions

	FWB		DET		DU		Difficulty		Control		Responsibility		Arousal		Valence	
	*M*	*s.e.*	*M*	*s.e.*	*M*	*s.e.*	*M*	*s.e.*	*M*	*s.e.*	*M*	*s.e.*	*M*	*s.e.*	*M*	*s.e.*
Free choice	22.3	0.44	16.6	0.5	24.4	0.5	1.96	0.12	5.63	0.1	5.99	0.11	2.16	0.1	4.78	0.1
Limited choice	22.2	0.47	16.9	0.45	23.9	4.71	3.15	0.14	3.6	0.12	3.61	0.14	2.99	0.12	4.21	0.11
No choice	22.4	0.49	17.9	0.52	24.0	0.55	2.78	0.16	2.83	0.13	3.07	0.15	2.5	0.12	4.26	0.11

### Hypothesis 2

We expected the manipulation to affect determinism beliefs but found moderate evidence against a difference in the mean determinism score between the three conditions (BF10 = 0.140, Fig. 3, Table 4). Thus we have found no difference in determinism beliefs between the three conditions.

### Exploratory analysis 1

We wanted to explore whether the manipulation affects dualism beliefs, but found strong evidence against a difference in mean determinism score between the three conditions (BF10 = 0.032, Fig. 3, Table 4). Thus we have found no difference in dualism beliefs between the three conditions.

### Exploratory analysis 2

We wanted to investigate how the experience of the manipulation game differed across conditions, by looking at perceived difficulty, control, responsibility, arousal, and valence. The descriptive statistics are shown in Table 4, and the results of the Bayesian ANOVAs and Bayesian Independent Samples *t* tests are shown in Table 5. Similar to the first study, we found that participants perceived the manipulation game as more difficult in the limited choice condition compared to the free choice condition. Here, the results indicated that participants perceived the no choice condition as more difficult than the free choice condition as well. Further, we found that participants felt most in control in the free choice condition, already less in control in the limited choice condition, and felt the least in control in the no choice condition. For responsibility, we found the same pattern of results as for the control question (free choice > limited choice > no choice). For arousal, we found that participants felt more aroused in the limited choice condition compared to the free choice condition, and less aroused in the limited choice condition compared to the no choice condition. Lastly, the results indicated that participants perceived the limited choice and no choice condition as more negative compared to the free choice condition.

**Table 5:** Analyses for control questions. We computed Bayesian ANOVAs and Bayesian Independent samples *t* tests for every control question, and the Bayes Factors (BF10) are reported

Test	Difficulty	Control	Responsibility	Arousal	Valence
ANOVA	> 100	> 100	> 100	> 100	94.85
free choice-limited choice	> 100	> 100	> 100	> 100	> 100
free choice-no choice	> 100	> 100	> 100	0.77	29.07
limited choice-no choice	0.52	> 100	3.37	5.29	0.13

### Exploratory analysis 3

We wanted to explore how agentive behavior in the manipulation game would differ across conditions. The Bayesian two-way ANOVA indicated decisive evidence for a difference in agentive behavior between the three conditions (BF10 > 100, Table 6), between the different time ranges (BF10 > 100), and for the interaction between condition and time range (BF10 > 100). Next, we performed post hoc two-way ANOVAs to compare the keypresses of the different conditions separately. For every combination of two conditions (free choice vs. limited choice; free choice vs. no choice; limited choice vs. no choice) we found decisive evidence for condition (BF10 > 100) and time range (BF10 > 100). For the interaction effect (condition * time), we found decisive evidence for the no choice and free choice condition (BF10 > 100), moderate evidence for the no choice and limited choice condition (BF10 = 4.50), but strong evidence against an effect was found for the free choice and limited choice condition (BF10 = 0.06). Thus we found that agentive behavior decreased with the amount of agency participants have in the manipulation game (free choice > limited choice > no choice). Over all conditions, we found that agentive behavior decreased when participants were proceeding further in the experiment, and this drop in agentive behavior was the strongest in the no choice condition compared to the free choice and limited choice condition.

**Table 6:** Descriptive statistics. The mean (*M*) and standard error (s.e.) of the total amount of keypresses in the manipulation game for every condition (free choice, limited choice, no choice), split by the time range (minute 1 vs 2 vs 3 vs 4 vs 5)

	Free choice		Limited choice		No choice	
	*M*	*s.e.*	*M*	*s.e.*	*M*	*s.e.*
1st min	50.40	2.05	60.30	3.71	42.80	4.38
2nd min	45.70	1.37	48.10	1.14	16.50	1.30
3rd min	41.90	1.28	46.70	1.15	15.10	2.05
4th min	39.30	0.92	47.80	2.60	13.10	1.48
5th min	38.70	1.30	44.30	1.89	11.20	1.38

We then combined the datasets of both studies and added experiment (1 vs 2) as a variable in a Bayesian three-way ANOVA (condition, time, experiment). For condition and time, we found the same results as for the second study alone. Further, there was evidence against the effect of the experiment (BF10 = 0.05), and against the three-way interaction (condition * time * experiment, BF10 < 0.01). Thus agentive behavior in the manipulation game did not differ across the two studies.

## Interim discussion

This second study showed that our experience-based manipulation of reduced agency over action and outcome had no impact on general beliefs in free will, determinism, or dualism. Thus we have found no transfer from the context to general beliefs. Further, we found that participants perceived less control and responsibility in the reduced agency conditions, and felt more negatively aroused. Lastly, our manipulation impacted participants’ agentive behavior in the game (i.e. data of keypresses to move forward, left and right).

## Discussion

In this current study, we developed a FWB manipulation technique by combining the social psychology and experimental philosophy approach, and by adding actual experience to the manipulation. As expected, we found that our experience-based manipulation decreased contextualized free will beliefs in the first study. Interestingly, while contextualized free will and dualism beliefs show no difference between the limited and no choice condition, other control variables (e.g. control, difficulty, and responsibility) show such a difference. Counter to our initial predictions, contextualized determinism beliefs were not affected by our manipulation. In the second study, although we expected our experience-based manipulation to also affect participants’ general beliefs outside the context due to the changing degree of agency (Aarts & van de Bos, 2011; Feldman, 2017; Lynn et al., 2014), this was not the case. In both studies, we found that participants perceived less control and responsibility in the reduced agency conditions, and felt more negatively aroused. Furthermore, we found in both studies that the manipulation had an impact on participants’ agentive behavior. Taken together, we found highly similar results in both studies, but the impact of our manipulation on context-specific FWB did not transfer to participants’ general beliefs.

### Experience vs abstract FWB manipulations

The existing abstract FWB manipulations have some potential concerns. These FWB manipulations used in social psychology and experimental philosophy increase the chance of potential demand effects, consist of complex philosophical arguments, and often employ hypothetical and far-fetched scenarios. An important asset of our newly developed manipulation technique is that we are the first to add actual experience to a FWB manipulation. Using this experience-based FWB manipulation, we addressed some of these potential concerns. First, using an experience-based manipulation, we did not have to explicitly tell participants that free will does not exist, or what a world without free will would look like. This is in contrast to methods commonly used in social psychology and experimental philosophy which explicitly call into question the existence of free will and might lead to demand effects. After all, participants read a text or scenario explicitly arguing about free will and related constructs (e.g. determinism), and afterward, fill in a questionnaire about free will related beliefs. Therefore, it is more likely that these previous results reflect such a demand effect rather than changes in a belief system that has been acquired over a lifetime (Tavernier, Wisniewski, Brass, in prep.). Thus using a more implicit manipulation, we could decrease this demand effect. Secondly, compared to these previous abstract manipulations, we did not implement complex philosophical arguments that could be difficult to understand by participants, instead, we used video games with simple instructions. Lastly, by adding experience to a scenario, they become less hypothetical and far-fetched, which makes them less reliant on the imagination of the participant. A potential disadvantage of our approach however could be that the use of an implicit experience-based approach elicits smaller effect sizes compared to an explicit approach.

### Effects on FWB do not generalize across contexts

In the first study, we found an effect on contextualized FWBs, which was measured using a modified version of the FWI. This indicates that the manipulation technique has an effect on contextualized free will beliefs. Furthermore, this effect of the manipulation is shown in the control questionnaires and in agentive behavior. Thus we can conclude that our manipulation is effective, as indicated by both questionnaires and behavior, which was not always the case with previous abstract text-based manipulation techniques (Monroe et al., 2017). Interestingly, we did not find an effect on contextualized determinism beliefs. This might be due to the nature of the determinism subscale. One could argue that the contextualized statements of the free will subscale (e.g. “Ertans always have the ability to do otherwise.”; “Ertans always have free will”) are more easily related to the experience-based manipulation compared to the contextualized determinism subscale (e.g. “Given the way things were at the Big Bang, there is only one way for everything to happen in Planet Erta after that”). However, we could make the same argument for the dualism subscale (e.g. “Each Ertan has a non-physical essence that makes that Ertan unique.”), where we did find an effect for dualism beliefs, making this explanation less likely. Another tentative explanation might be that the impact of a FWB manipulation is just stronger on free will beliefs compared to determinism beliefs. This is also what has been found in previous literature (Genschow et al., 2021).

In the second study, we found no effect on general FWBs, which was measured using the regular FWI. This suggests an interesting interpretation of previous results. In previous studies, these manipulation checks (i.e. impact on general FWBs) often work (Genschow et al., 2021). However, when we remove demand effects when using an experience-based manipulation, this manipulation check does not work anymore. Therefore, it is important for future studies to control for this demand effect much more strongly. Importantly, if these successful manipulation checks in literature are mostly driven by demand effects, a lack of impact on downstream behavior is actually expected.

Based on our results in the second study, one might argue that this conclusion (i.e. no effect on general FWBs) is based on a null finding. However, we adequately powered and pre-registered this study, and found evidence for the null hypothesis in our analyses. Thus we can conclude that no transfer occurred from contextualized to general FWBs. A tentative explanation to why we did not find this transfer is the difference between state and trait beliefs. Our manipulation which consists of an experience of reduced agency over outcome and action has an impact on FWB within the specific context of that manipulation. This is the impact on a state belief, i.e. a belief of a person given that they are in this specific situation. Contrarily, this manipulation had no impact on general FWB. This is the absence of an impact on a trait belief, i.e. a hard-held belief given the person (Steyer et al., 1999). Here we could argue that it is quite hard to change people’s hard-held trait beliefs by short-term exposure to a reduced agency experience (Wood & Denissen, 2014). However, we did not expect that this short-term exposure would completely change a complex system of trait beliefs, but rather we expected a stronger priming effect of our manipulation on trait FWB compared to previous methods, because of the direct experience to reduced agency instead of merely reading a theoretical argument.

Furthermore, another possible contribution to the lack of transfer could be due to the third-person perspective of our manipulation. It could be that participants less identify with a third-person perspective view in the manipulation, and therefore distance their own beliefs from the beliefs of the virtual agent. Besides, it could be argued that because of this third-person perspective we are investigating free will belief attribution to this virtual agent rather than actual belief change of the participant. However, participants are still experiencing the reduced agency themselves, thus it seems highly doubtable that the effect is merely dependent on belief attribution. Future research needs to be done to understand the difference between state vs trait beliefs and first vs third perspective in relation to FWB manipulations.

Moreover, the lack of transfer to general FWB could also be due to the fact that participants are still introduced to *Planet Erta* in the second study. Here, we had the goal to use the exact same procedure for both studies, and solely change the questionnaire from studying contextualized to general FWB. Nevertheless, it could be that introducing participants to another “world” (i.e. *Planet Erta*) reduces the impact of the manipulation on general FWB. Thus it could be interesting for future studies to investigate whether an experience-based manipulation which is set in a more realistic context might translate better to changes in general FWB. Overall, we see no evidence for a transfer from contextualized to generalized FWB. However, to draw strong conclusions, further studies with improved designs would be required. Here, we suggest future studies to create an experience-based FWB manipulation in which participants act from a first-person perspective. Furthermore, we suggest creating a context which is more realistic and relates to the daily life of the participant. Additionally, we suggest this experience to be offline rather than online, and allowing the measurement of downstream processes (e.g. behavior and cognition).

### The case for contextualized FWB manipulations

Although our contextualized FWB manipulation does not impact general FWB, the approach might nevertheless be useful for future research on the relation between FWB and downstream processes. First, the question arises whether it is realistic to change long-held beliefs with a relatively brief manipulation. While previous manipulations revealed a reliable decrease of free will beliefs in questionnaires, such effects might have been partly due to demand effects. Our findings suggest that it is possible to change contextualized free will beliefs with an implicit manipulation that is presumably much less susceptible to demand effects. Besides, even if it would be possible to substantially change long-held beliefs in free will, this might raise severe ethical issues, as we do not yet know what the behavioral impact of this belief change might be. Limiting ourselves to contextualized manipulations addresses both of these potential concerns. Therefore, we believe it could be interesting to study FWB-behavior interactions without attempting to change hard-held trait beliefs regarding free will.

Using a contextualized manipulation, an encapsulated model of beliefs can be created in which the behavioral impact could be investigated. Here, participants immerse in a context where they have reduced agency over actions and outcomes. Then, implicit behaviors and attributions related to FWB could be measured within this context, without having to change their beliefs outside of it. While we can study the effects of beliefs on behavior in this encapsulated context, there are of course disadvantages as well. Most importantly, we cannot claim that our findings generalize to everyday behavior. Nevertheless, we can still investigate basic psychological processes, like how beliefs and behavior interact. This will rely on creating powerful contextual cues however, since this approach requires participants to immerse themselves in the presented scenarios. For this purpose, Virtual Reality (VR) might provide a powerful tool. Previous research has already shown the high presence and immersion that can be provided using VR, and the increasing use of VR in different domains of research has shown some advantages over the traditional experimental settings (Kozlov & Johansen, 2010; Slater & Sanchez-Vivez, 2016; Wilson & Soranzo, 2015). Compared to video games, participants report even higher presence and immersion while being in VR, which is described as the feeling of leaving reality and being transported to another world where they perceive objects and situations as real (Slater & Sanchez-Vivez, 2016). This level of immersion and presence is highly advantageous for creating a scenario in which decreasing agency is experienced. An additional advantage of VR is that certain measurements can be used, such as eye- and movement tracking, to capture the downstream processes of an experience-based FWB manipulation. Taken together, the current study shines a new light on previous research and suggest a way forward for studying belief-behavior interactions in the context of free will.

## Data Availability

All materials, data and code are available on the Open Science Framework (https://osf.io/xh36s/).The pre-registrations can also be found on the OSF (Study 1: https://osf.io/bsf8w; Study 2: https://osf.io/2rucd).
